# Treatment Outcomes of Tuberculosis in the Eastern Cape: Clinical and Socio-Demographic Predictors from Two Rural Clinics

**DOI:** 10.3390/ijerph22121804

**Published:** 2025-11-29

**Authors:** Evidence L. Nxumalo, Ncomeka Sineke, Ntandazo Dlatu, Teke Apalata, Lindiwe Modest Faye

**Affiliations:** 1 Department of Laboratory Medicine and Pathology, Faculty of Medicine and Health Sciences, Walter Sisulu University, Private Bag X1, Mthatha 5100, South Africa; 230026575@mywsu.ac.za (E.L.N.); 209101237@mywsu.ac.za (N.S.);; 2 Department of Public Health, Faculty of Medicine and Health Sciences, Walter Sisulu University, Private Bag X1, Mthatha 5100, South Africa; ndlatu@wsu.ac.za

**Keywords:** tuberculosis, treatment outcomes, eastern cape, South Africa, GIS, HIV co-infection

## Abstract

**Background**: Tuberculosis (TB) remains a leading cause of morbidity and mortality, with South Africa among the highest-burden countries. The Eastern Cape is particularly affected due to poverty, HIV co-infection, and weak health systems. Understanding treatment outcomes and their determinants is required to achieve the WHO End TB Strategy targets. The objective of this study was to examine treatment outcomes for tuberculosis (TB) in both rural and urban clinics within the Eastern Cape Province. We aimed to identify the socio-demographic, clinical, and geographic factors that influence treatment success or failure. We included simple geographic visualisations comparing treatment outcomes between the two participating clinics to inform the development of targeted interventions aimed at enhancing TB control efforts. **Methods**: A retrospective cohort study of 385 TB patients treated at two public clinics in the Eastern Cape (2020–2024) was conducted. Socio-demographic, clinical, and geographical data were extracted from records. Outcomes were classified using WHO and South African National TB Programme guidelines. Logistic regression identified predictors of success, and spatial analysis mapped treatment outcomes. **Results**: The mean patient age was 40.6 years; 69.1% were HIV-positive, and 89.9% had pulmonary TB. The overall treatment success rate was 63.8%, below the WHO target of ≥85%. Pulmonary TB was independently associated with greater odds of success (aOR = 2.86, 95% CI: 1.23–6.65), while older age predicted poorer outcomes (aOR = 0.98, 95% CI: 0.963–0.998). HIV status and socioeconomic variables were not independently associated after adjustment, although poverty and unemployment were widespread. Spatial mapping showed clustering of poor outcomes in specific clinics, highlighting geographic and health system disparities. **Conclusions**: TB treatment outcomes in the Eastern Cape remain unsatisfactory. Older patients and those with extrapulmonary TB face higher risks of unfavourable outcomes, underscoring the need for closer monitoring and adherence support. Integrated TB/HIV care, social protection, and geographically targeted interventions are essential to strengthen health systems and reduce inequalities.

## 1. Introduction

Tuberculosis (TB) remains one of the leading causes of morbidity and mortality globally, with an estimated 10.8 million new cases, including 6.0 million women and 1.3 million children [1]. South Africa carries one of the highest burdens of TB worldwide, accounting for nearly 3% of the global caseload, with the Eastern Cape Province identified as a high-incidence region [2]. The dual epidemics of TB and HIV further complicate disease control, with HIV co-infection substantially increasing the risk of progression from latent infection to active TB disease [3,4]. Despite advances in diagnosis and treatment, achieving the World Health Organization (WHO) End TB Strategy target of a 90% reduction in TB incidence and 95% reduction in mortality by 2035 remains challenging, particularly in resource-limited rural settings [5]. Treatment outcomes are a critical measure of the effectiveness of TB control programmes, with WHO recommending a treatment success rate of at least 85% as a benchmark for quality of care [6]. However, South Africa’s reported treatment success rates remain below this threshold, varying across provinces and between rural and urban health systems [6]. Factors such as age, sex, socioeconomic status, HIV co-infection, drug resistance, and previous TB history have been associated with treatment outcomes, yet these associations often vary by context [7]. Spatial heterogeneity in outcomes has been reported, suggesting that geographic and health system factors contribute to disparities in treatment success and failure [8,9].

The Eastern Cape Province presents unique challenges for TB control due to high levels of poverty, unemployment, and limited health system resources. Rural areas are characterised by poor healthcare access, long travel distances, and inadequate infrastructure, which may exacerbate risks of follow-up loss, treatment failure, and mortality [10]. Under appropriate circumstances, geographic information can help highlight differences in outcomes between health service locations; however, with only two clinics included, the study could only generate descriptive, location-based visualizations rather than spatial mapping to visualize spatial patterns, identify high-risk areas, and guide targeted interventions to strengthen TB care.

This study aimed to map TB treatment outcomes in the Eastern Cape Province and identify the socio-demographic, clinical, and health system factors influencing success and failure, to inform targeted interventions and improve TB control strategies in both rural and urban contexts.

## 2. Materials and Methods

This retrospective cohort study focused on patients diagnosed with both drug-susceptible and drug-resistant tuberculosis (TB) who initiated treatment at two selected public health clinics in the Eastern Cape Province, South Africa, from January 2020 to December 2024. The clinics were intentionally chosen to represent both rural and peri-urban settings within the province. The selection criteria included: (i) the availability of complete TB treatment records from 2020 to 2024, (ii) the capacity to manage both drug-susceptible and drug-resistant TB cases, and (iii) accessibility for periodic data verification. Initially, 452 patients were screened. Among these, 67 patients (14.8%) were excluded due to missing treatment outcome information or incomplete demographic and clinical records. As a result, the final retrospective cohort consisted of 385 patients with complete data for analysis. Out of the 412 patient records initially reviewed, 27 records (6.6%) were excluded because of missing outcome information or incomplete demographic and clinical data. This left a total of 385 patients with complete datasets available for analysis. While the percentage of excluded records was relatively small, this exclusion may have slightly affected the representativeness of the treatment outcomes, as mentioned in the study’s limitations.

### 2.1. Variables

#### 2.1.1. Dependent Variable

The primary outcome of interest was TB treatment outcome, defined according to WHO and South African National TB Programme guidelines [11,12]. Outcomes were categorised as successful (cured or treatment completed) or unsuccessful (treatment failure, loss to follow-up, or death). Patients transferred out, relocated, or still on treatment were coded as censored and excluded from regression analyses. Treatment outcomes were analysed for both drug-susceptible (DS-TB) and drug-resistant TB (DR-TB) cases. The overall treatment success rate was 63.8% (67.5% among DS-TB patients and 58.2% among DR-TB patients).

#### 2.1.2. Independent Variables

Socio-demographic variables included age (in years), sex, education level (none, primary, secondary, tertiary), occupation (unemployed, student/pensioner/grant, government/private sector, or other), and income status (salary/wages, casual/other, self-employed, or no income). Employment history was also recorded. The “other” category captured informal, temporary, or subsistence economic activities such as street vending, small-scale farming, or domestic work, which are common in rural and peri-urban Eastern Cape settings. This distinction was retained to reflect the informal labour segment, which may influence treatment adherence through unstable income or irregular work patterns.

Clinical variables comprised HIV status (positive/negative), TB type (pulmonary or extrapulmonary), previous TB history (ever treated for TB in the past), patient category (new or retreatment case per South African National TB Programme classification), comorbidities (diabetes mellitus, hypertension, HIV, kidney disease, epilepsy; yes/no), drug resistance type, and DR-TB grouping. The kind of resistance variable reflected specific laboratory-identified resistance profiles (e.g., rifampicin-resistant, MDR, pre-XDR, XDR, or isoniazid-monoresistant). In contrast, DR-TB grouping was a programmatic classification used for clinical management and reporting, categorizing patients as drug-susceptible TB, mono- or poly-drug-resistant TB, or multidrug/extensively drug-resistant TB (MDR/XDR-TB). Maintaining both variables enabled analysis of molecular resistance patterns alongside broader epidemiological groupings, allowing differentiation between historical exposure and current clinical status.

Behavioural variables included smoking and alcohol use, analysed as risk factors influencing treatment adherence and immune function. Social grant dependency was treated as a proxy for social protection and economic vulnerability, reflecting reliance on government assistance rather than formal employment. Collectively, these behavioural and social determinants were considered potential indirect influences on TB treatment outcomes.

### 2.2. Data Analysis

Data were entered into Microsoft Excel and analyzed using Stata 17 (StataCorp, College Station, TX, USA) and Python (version 3.11) for advanced statistical modelling and spatial visualization. Descriptive statistics were first computed to summarise socio-demographic, clinical, and treatment outcome variables. Continuous variables, such as age, were reported as means with standard deviations (SD) and medians with interquartile ranges (IQR), depending on distribution. Categorical variables were presented as frequencies and proportions. Bivariate analyses were conducted to examine the associations between independent variables and treatment outcomes (success vs. failure). The chi-square test (χ^2^) or Fisher’s exact test (for small cell sizes) was used for categorical variables, while Student’s *t*-test was applied for continuous variables. Statistical significance was set at *p* < 0.05. To identify independent predictors of treatment success, multivariable logistic regression was conducted. A simplified model was initially constructed to include key socio-demographic and clinical variables identified from bivariate analysis. A reduced model was then fitted after testing for multicollinearity using variance inflation factors (VIF), retaining only variables that contributed significantly to model fit. Adjusted odds ratios (aOR) with 95% confidence intervals (CIs) and *p*-values were reported. Basic geographic visualisations were generated using the two clinic coordinates (latitude and longitude). These visual outputs were intended solely to compare the distribution of treatment outcomes between the two clinics. Outcomes were mapped using scatter and hexbin density plots to illustrate the geographic distribution of treatment success and failure. HIV prevalence and socioeconomic indicators (e.g., no income) were also spatially mapped to assess overlapping vulnerabilities.

## 3. Results

### 3.1. Descriptive Characteristics

A total of 385 patients were included in the analysis. The mean age was 40.6 years (SD 12.6), with a median of 40 years (IQR 31–49). Gender distribution was balanced, with 198 (51.4%) male and 187 (48.6%) female participants. More than half of the cohort (58.2%) reported no income, and 54.5% had attained secondary education. The majority were unemployed (46.5%) or dependent on pensions and social grants (24.9%). Clinically, pulmonary TB (PTB) predominated (89.9%), with extrapulmonary TB (EPTB) accounting for 10.1% of cases. HIV co-infection was observed in 266 patients (69.1%), and in terms of drug resistance, rifampicin resistance (RR) accounted for 34.3% of cases. At the same time, the remainder were classified as MDR, pre-XDR, XDR, or isoniazid-monoresistant. Treatment outcomes revealed that 91 patients (23.6%) were cured, 69 (17.9%) completed treatment, 36 (9.4%) were lost to follow-up (LTFU), 21 (5.5%) experienced treatment failure, and 22 (5.7%) died. A further 119 patients (30.9%) were transferred or discharged, while 27 (7.0%) remained under treatment at the time of analysis. Among definitive outcomes, the overall treatment success rate (cured or completed) was 63.8%, as shown in Table 1.

**Figure 1:**
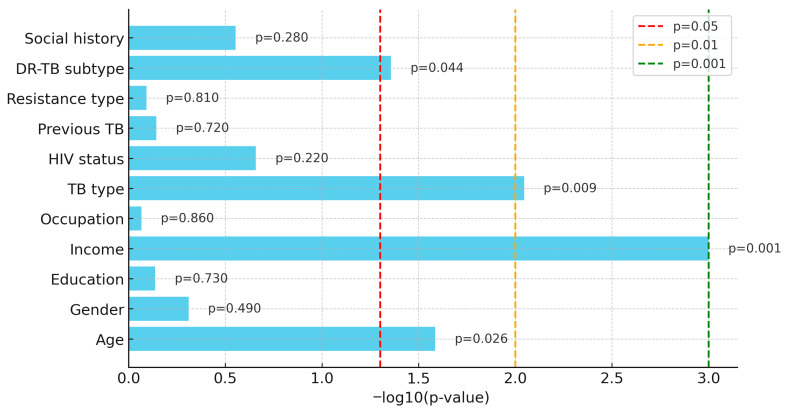
Bivariate Test: Significance of associations with treatment success.

Table 2 and Figure 1 illustrate the significance of associations between patient characteristics and TB treatment success. The x-axis (Figure 1) displays −log10(*p*-values), where higher values indicate more substantial evidence of association. Vertical dashed lines mark conventional significance thresholds (*p* = 0.05, 0.01, 0.001). Income (*p* < 0.001), TB type (*p* = 0.009), and age (*p* = 0.026) were significant predictors, with DR-TB subtype showing a weaker effect (*p* = 0.044). It is important to note that *p*-values reflect whether a relationship is statistically significant compared to the specified alpha level. Still, they do not quantify the strength of the association. Instead of using the absolute value of the *p*-value, odds ratios and confidence intervals were used to interpret the magnitude of the effect. Patients reporting no income were more likely to achieve successful outcomes, pulmonary TB was associated with better outcomes compared to extrapulmonary TB, and older patients were more likely to experience unsuccessful outcomes. Gender, education, occupation, HIV status, previous TB history, resistance type, and social history were not significantly associated with treatment outcomes (*p* > 0.05). These findings informed a multivariable regression analysis, where age and TB type remained significant, while the effect of income was partially explained by collinearity.

Table 3: Multivariable logistic regression identified pulmonary TB and younger age as independent predictors of treatment success. Patients with pulmonary TB were nearly three times more likely to achieve successful outcomes compared to those with extra-pulmonary disease (OR = 2.86, 95% CI 1.23–6.65, *p* = 0.015). Increasing age was associated with reduced odds of treatment success, with each additional year lowering the likelihood of a favourable outcome by approximately 2% (OR = 0.98, 95% CI 0.963–0.998, *p* = 0.026). In the simplified model, socioeconomic indicators such as no income (OR = 5.98, *p* < 0.001) and formal employment (OR = 4.43, *p* = 0.007) appeared strongly predictive of success. Still, these effects attenuated in the reduced model, suggesting collinearity with other clinical factors. Gender, HIV status, retreatment, and drug resistance type were not significantly associated with treatment outcome in the adjusted models. These findings highlight the greater vulnerability of older patients and those with extra-pulmonary TB, underscoring the need for enhanced monitoring and tailored support for these groups.

### 3.2. Bivariate Associations

Bivariate analyses revealed significant associations between age, income, and type of TB and treatment success. Successful patients were significantly younger than unsuccessful patients (mean 39.8 vs. 42.5 years, *p* = 0.026). Patients reporting no income were more likely to achieve successful outcomes (*p* < 0.001). PTB was strongly associated with success compared to EPTB (*p* = 0.009). There was also variation by DR-TB subtype, with poorer outcomes observed among XDR cases (*p* = 0.044). No significant differences were observed in terms of gender, education, HIV status, occupation, or social history.

### 3.3. Multivariable Regression

**Figure 2:**
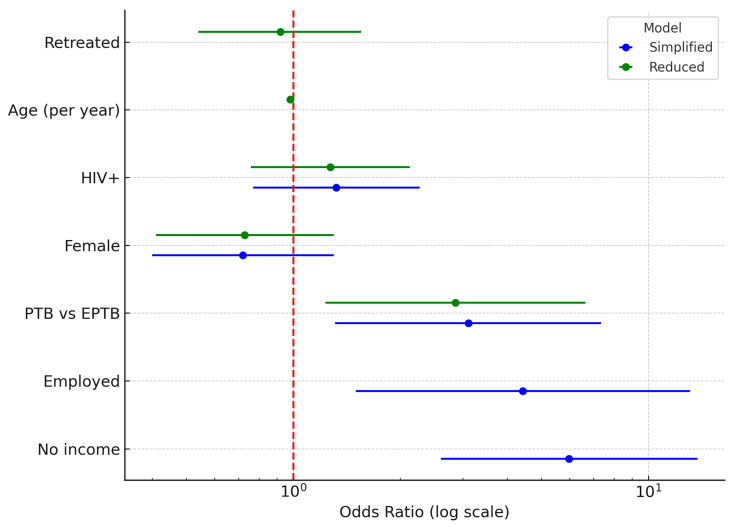
Comparative forest plot: Simplified vs. reduced models.

In the simplified model, no income (adjusted OR = 5.98, 95% CI 2.60–13.74; *p* < 0.001), employment in government or private sector (OR = 4.43, 95% CI 1.50–13.10; *p* = 0.007), and PTB (OR = 3.11, 95% CI 1.31–7.35; *p* = 0.010) were significantly associated with treatment success (Figure 2).

After adjusting for multicollinearity in the reduced model, PTB remained a strong independent predictor of success (OR = 2.86, 95% CI 1.23–6.65; *p* = 0.015), and increasing age was associated with reduced odds of success (OR = 0.98 per year, 95% CI 0.963–0.998; *p* = 0.026). Gender, HIV status, retreatment status, resistance type, and DR-TB grouping were not significantly associated with treatment outcome.

**Figure 3:**
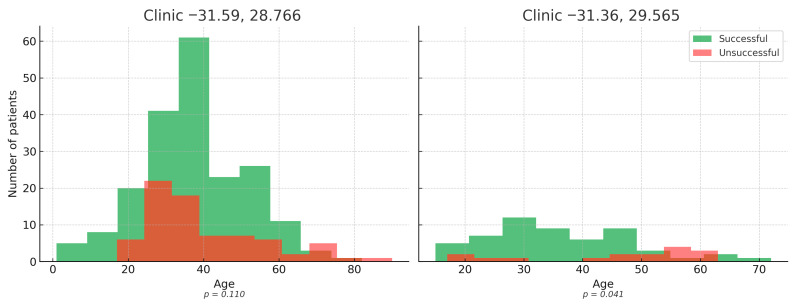
Age distribution by treatment success across clinics.

Age distribution analyses demonstrated that unsuccessful outcomes were consistently skewed toward older patients. In the combined cohort, patients with unsuccessful outcomes were significantly older than those with successful outcomes (*p* = 0.026) (Figure 3). Stratified analyses by clinic revealed the same trend, although within-site differences did not reach statistical significance, likely due to limited sample size. The consistent direction of effect across both clinics supports the robustness of age as a predictor of poor outcomes, underscoring the need for enhanced monitoring and tailored support for older TB patients.

Age distribution of patients by treatment outcome. The combined analysis (left panel) reveals that patients with unsuccessful outcomes were significantly older than those with successful outcomes (*t*-test, *p* = 0.026). Stratified analyses by clinic (right panel) demonstrated the same trend, although within-site differences were not statistically significant, likely due to smaller sample sizes. The consistent pattern across both clinics reinforces age as a predictor of poor outcomes, highlighting older patients as a high-risk group requiring targeted support.

**Figure 4:**
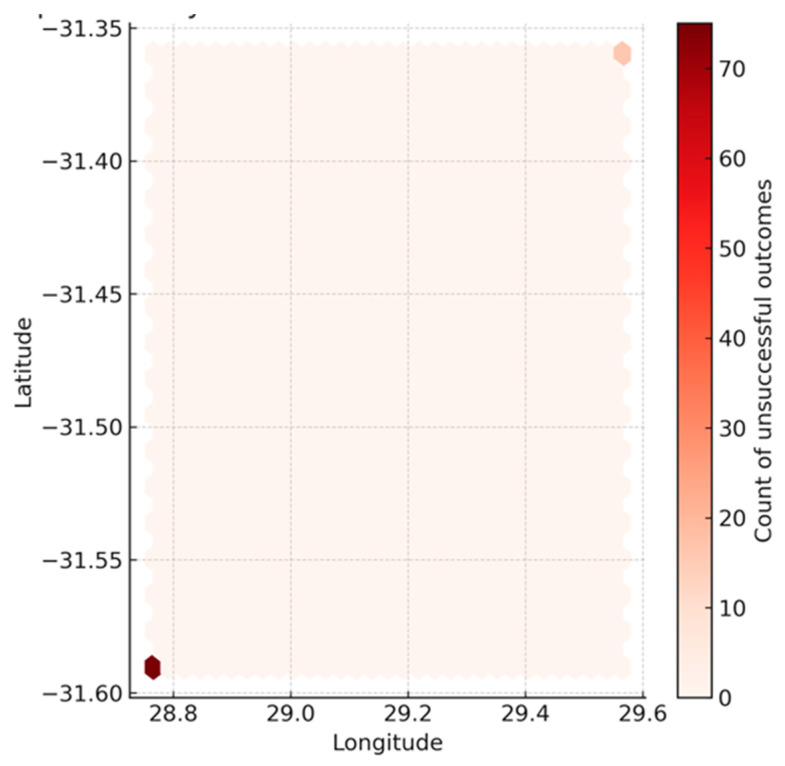
Descriptive geographic visualisation of unsuccessful TB treatment outcomes at the two study clinics. Notes: Each hexagon represents aggregated cases at the corresponding clinic coordinate.

The hexbin density map illustrates the spatial distribution of unsuccessful TB treatment outcomes (lost to follow-up, failed, or died), with colour intensity representing the number of cases at each clinic coordinate (Figure 4). Two distinct clusters are visible, corresponding to the two clinics in the dataset. The two coloured hexagons correspond to the two clinic locations in the dataset, with each hexagon representing a cluster of patients treated at that site; the colour intensity reflects the number of unsuccessful outcomes recorded. Clinic A (bottom-left, longitude ≈ 28.8, latitude ≈ −31.59) exhibited the highest density of unsuccessful outcomes (dark red, ~70+ cases), while Clinic B (top-right, longitude ≈ 29.6, latitude ≈ −31.35) recorded fewer events (lighter red, ~10–20 cases). These findings indicate that unsuccessful outcomes are concentrated in two geographic sites, with one clinic carrying a much larger absolute burden. However, when adjusting for patient volume, the proportions of unsuccessful outcomes were not statistically different between the sites (Fisher’s exact test, OR = 1.30, *p* = 0.45), suggesting that the apparent difference reflects case load rather than actual variation in treatment success rates.

**Figure 5:**
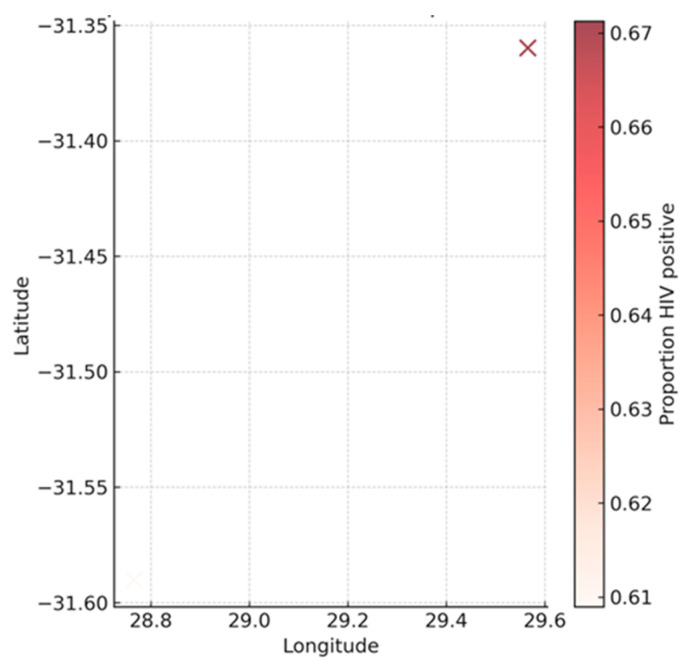
Geographic comparison of HIV prevalence between the two clinics. Notes: Clinic coordinates are marked with “X”.

Figure 5 map describes the spatial distribution of HIV prevalence across the two clinics, with colour intensity indicating the proportion of patients who were HIV positive at each site. Clinic A (bottom-left, longitude ≈ 28.8, latitude ≈ −31.59) recorded a prevalence of approximately 61%, shown in a lighter shade, while Clinic B (top-right, longitude ≈ 29.6, latitude ≈ −31.35) recorded a higher prevalence of around 67%, indicated by a darker shade. The “X” symbols mark the geographic coordinates of the two clinic locations, each representing the cluster of patients treated at that site. Although both clinics indicate a similar altitude, they differ significantly in longitude, and their relative east–west separation is what enables spatial distinction in our maps. These results show that HIV prevalence is uniformly high across both clinics, with only minor variation in levels, underscoring the importance of integrated TB/HIV management in this population.

**Figure 6:**
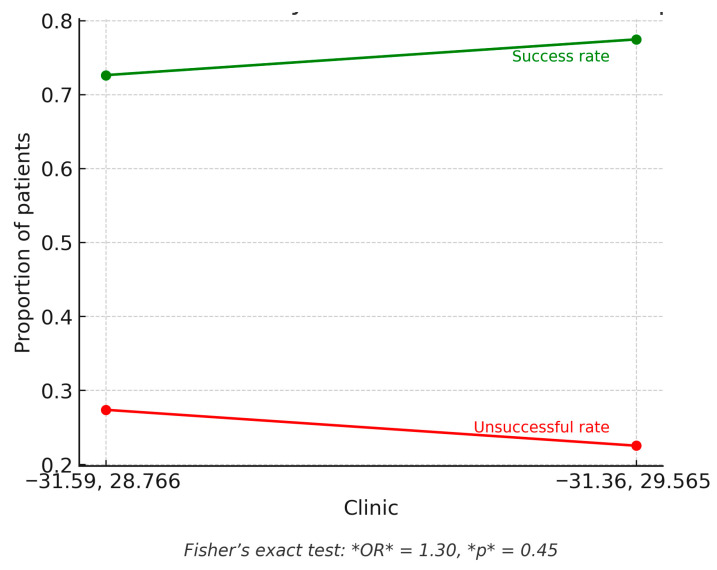
Treatment outcomes by clinic. Notes: This figure compares the proportions of successful and unsuccessful treatment outcomes between the two clinics.

Figure 6 illustrates treatment outcomes by clinic, accompanied by a statistical comparison. The green line shows the proportion of patients achieving treatment success (cured or completed), while the red line shows the proportion with unsuccessful outcomes (lost to follow-up, failure, or death). Clinic B (longitude ≈ 29.565) recorded a slightly higher success rate (~78%) compared to Clinic A (~73%), but the difference was not statistically significant (Fisher’s exact test: OR = 1.30, *p* = 0.45.

## 4. Discussion

This study analysed TB treatment outcomes from two rural clinics. It provided simple geographic visualisations of clinic-level differences in the rural Eastern Cape, identifying socio-demographic and clinical predictors of success. Similarly, Reddy et al. (2025 highlighted several factors that impede treatment success, including access to quality healthcare, financial well-being, and education [13]. The overall treatment success rate of 63.8% among definitive outcomes is lower than both the WHO target of ≥85% and South Africa’s reported national average, which has ranged from 70% to 78% in recent years [1,12]. Comparably, a study in Botswana reported a treatment rate between 88–91%, while another from Nigeria found that 84% HIV/TB co-infected patients were successfully treated for TB [4]. Moreover, although transfer-out and moved cases are not classified as lost to follow-up under South African NTP rules and are therefore omitted from outcome calculations, the large proportion observed in this study (30.9%) is noteworthy. A large number of unverified outcomes may conceal the full magnitude of negative results and reflect underlying issues such as patient mobility, inter-facility referral patterns, and poor cross-facility data integration. This limitation affects the comprehensiveness of programmatic evaluation and emphasises the necessity for improved tracking methods for transferred patients. Our study findings highlight persistent gaps in TB control in rural areas with high HIV burden and socioeconomic deprivation.

### 4.1. Predictors of Treatment Outcomes

Younger age and pulmonary TB emerged as consistent predictors of success. The adverse effect of increasing age mirrors previous studies showing that older patients experience higher mortality, treatment failure, and loss to follow-up, often due to comorbidities, reduced adherence, and socioeconomic vulnerability [7]. Faye et al. (2023) emphasised how age increase is associated with decreased probability of completing treatment [6]. In agreement with this, a study conducted in Nigeria to assess TB patient profiles and predictors of treatment success reported that older (≥50 years) age was one of the variables linked to unsuccessful outcomes [14]. The strong association between PTB and favourable outcomes likely reflects the relative ease of diagnosis and monitoring compared to EPTB, which is frequently underdiagnosed and more difficult to manage [15]

Although HIV prevalence was high (69%), HIV status was not independently associated with treatment outcomes after adjustment. This may reflect improvements in HIV/TB integration and ART rollout, though HIV/TB co-infected patients remain at risk of poorer outcomes, particularly mortality [3]. The 2016 WHO ART guidelines, which transitioned from treatment initiation based on CD4 count thresholds to a treat-all strategy, may be a significant factor in improved treatment outcomes; however, access remains a key influence [16]. Socioeconomic indicators, especially income and employment, were predictive in unadjusted models but attenuated in multivariable models, suggesting indirect pathways through health-seeking behaviour, comorbidity, and treatment adherence. Interestingly, patients without income sometimes had better outcomes, a counterintuitive finding that may be explained by their reliance on social grants and greater engagement with public health services, a phenomenon also reported in other South African cohorts [17]. Our findings align with a study conducted in Uganda, which emphasises that low socioeconomic status is associated with poor treatment outcomes and the need for cross-sectoral mitigation strategies [18]. This underscores the importance of contextual vulnerability where poverty indirectly affects outcomes through late presentation, comorbidities, and weaker social support.

### 4.2. Spatial and Geographical Patterns

Given that only two clinic locations were available, the geographic figures presented are descriptive visual comparisons. They illustrate differences in case burden between the two clinics. Visualisations nonetheless suggested localised high-risk pockets, aligning with evidence that TB outcomes vary geographically depending on healthcare access, socioeconomic gradients, and health system performance [8]. Internationally, geospatial studies consistently demonstrate that TB clusters in disadvantaged communities. A systematic review of 79 studies identified low socioeconomic conditions (39%), high population density (17%), and climate variability (15%) as the most common predictors of high-risk areas [19]. Our findings fit this global framework, with poverty and unemployment compounding the effects of HIV prevalence and weak rural health systems.

### 4.3. Global Comparisons

The Eastern Cape findings align with global literature, while also highlighting the unique features of South Africa’s epidemic. Taiwan, for example, achieved a 62% decline in TB incidence between 2005 and 2023 through strong political commitment, universal drug-resistance diagnostics, and innovative surveillance [20]. By contrast, Pakistan experienced a rising TB incidence until 2018, attributed to climate and population density, followed by a modest decline [21]. In Beijing, TB incidence was shaped by socioeconomic and healthcare infrastructure predictors [22]. In high-income contexts such as the United States, TB persists in poverty-stricken or marginalised communities, despite overall low prevalence [23]. Within South Africa, the overlapping syndemic of TB and HIV characterizes the local context. A systematic review indicated that underserved populations in South Africa have a four-times higher TB prevalence among HIV-negative individuals and a 31-times higher prevalence among PLWH compared to national averages [24]. This aligns with our observation that unemployment, low income, and low education worsen health outcomes.

### 4.4. Implications for Policy and Practice

Multivariable analysis identified pulmonary TB and younger age as independent predictors of treatment success, consistent with previous evidence that extrapulmonary disease poses greater diagnostic and management challenges [6,25]. Age was inversely associated with success, with older patients more likely to experience unfavourable outcomes, echoing studies that link advanced age to higher rates of default, mortality, and complications in TB treatment [26]. The finding that patients with no income were more likely to achieve success is counterintuitive. It may reflect the influence of social grants or higher engagement with public health support services, a phenomenon noted in other South African cohorts [27].

Taken together, these findings emphasise the multifactorial determinants of TB outcomes in rural South Africa. At the clinical level, older patients and those with extrapulmonary TB require enhanced monitoring, adherence support, and closer follow-up. At the social level, interventions such as transport vouchers, food parcels, and social protection schemes could mitigate barriers to treatment success. At the governance level, clinic-level data monitoring and spatially sensitive TB surveillance systems are crucial for identifying and targeting geographic areas with poor outcomes.

Globally, our findings reinforce that TB outcomes are shaped by a convergence of biomedical factors (age, TB type, HIV status, drug resistance) and structural determinants (poverty, unemployment, low education, access to care). While Asian studies underscore the importance of climate and migration, and high-income settings emphasise hidden poverty clusters, the South African context remains uniquely defined by the TB–HIV syndemic and entrenched socioeconomic inequities. Addressing these requires integrated approaches that combine clinical governance and community engagement with targeted socioeconomic interventions to move closer to WHO treatment targets.

### 4.5. Strengths and Limitations

A key strength of our study lies in the integration of clinical, socio-demographic, and spatial variables, allowing a comprehensive assessment of TB treatment outcomes in a high-burden rural setting. By including income, employment, education, HIV status, TB type, and retreatment history, the analysis captures the syndemic nature of TB in South Africa. The use of routinely collected clinical data also enhances the study’s real-world relevance for programme planning and policy. Nevertheless, several limitations must be acknowledged. The cross-sectional design limits causal inference: longitudinal studies are required to understand treatment trajectories over time. Missing data and the exclusion of patients transferred out or still on treatment may have introduced selection bias, potentially underestimating poor outcomes. Geographic visualisation was limited by the availability of only two clinic coordinates. Consequently, the figures provided do not constitute spatial analysis; they display treatment outcomes by clinic. Finally, generalizability is limited, as the findings are based on data from two clinics in the rural Eastern Cape and may not accurately reflect patterns across South Africa’s diverse settings. Despite these limitations, the study provides robust, context-specific evidence that socioeconomic deprivation, HIV burden, and health system weaknesses converge to shape TB outcomes. These insights are crucial for designing governance-driven, community-based interventions that promote equity, enhance treatment outcomes, and address the complex interplay between clinical and social determinants of TB.

## 5. Conclusions

According to the guidelines set by the WHO and the South African National TB Programme, patients who were transferred to another facility or moved were not classified as lost to follow-up. As a result, they were excluded from the total count when calculating definitive treatment outcomes. The reported success rate of 63.8% represents the percentage of patients who were either cured or completed their treatment among those with known outcomes, excluding cases that were transferred, relocated, or still ongoing at the time of reporting. Clinic-level visual comparisons indicated differences in the burden of poor treatment outcomes between the two sites, alongside similarly high levels of HIV prevalence. Although the study could not establish any causal relationship between these patterns, their convergence suggests that strengthened, integrated TB–HIV service delivery may be beneficial for both clinics. This observation warrants further investigation through longitudinal and programmatic studies.

## Figures and Tables

**Table 1:** Descriptive Characteristics and Tuberculosis (TB) Treatment Outcomes of the Study Population (n = 385).

Variable	Category	N	%
Age (years)	Mean ± SD	40.6 ± 12.6	—
	Median (IQR)	40 (31–49)	—
Gender	Male	198	51.4
	Female	187	48.6
Education	No schooling	18	4.7
	Primary	101	26.2
	Secondary	210	54.5
	Tertiary	56	14.5
Income	Salary/Wages	42	10.9
	Casual/Other	31	8.1
	No income	224	58.2
	Self-employed/Other	88	22.9
TB type	Pulmonary (PTB)	346	89.9
	Extrapulmonary (EPTB)	39	10.1
HIV status	Positive	266	69.1
	Negative	119	30.9
Treatment outcome	Cured	91	23.6
	Completed	69	17.9
	Lost to follow-up (LTFU)	36	9.4
	Failed	21	5.5
	Died	22	5.7
	Transferred/Moved out	119	30.9
	Still on treatment	27	7.0
Combined outcome categories	Successful (Cured + Completed)	160	63.8 ^1^
	Unsuccessful (LTFU + Failed + Died + Transferred + Still)	91	36.2 ^1^
Treatment outcome by drug-resistance status	Successful—DS-TB	—	67.5 ^2^
	Successful—DR-TB	—	58.2 ^2^
	Unsuccessful—DS-TB	—	32.5 ^2^
	Unsuccessful—DR-TB	—	41.8 ^2^

^1^ Calculated among patients with definitive outcomes (excluding those transferred or still on treatment). ^2^ Sub-analysis stratified by drug-susceptible (DS-TB) and drug-resistant (DR-TB) cases, consistent with WHO 2022 End TB Strategy reporting.

**Table 2:** Bivariate associations between patient characteristics and treatment success.

Variable	χ^2^/t	*p*-Value	Sig.
Age (years)	t = −2.24	0.026	*
Gender	χ^2^ = 0.47	0.490	ns
Education	χ^2^ = 1.29	0.730	ns
Income	χ^2^ = 14.7	<0.001	***
Occupation	χ^2^ = 0.82	0.860	ns
TB type (PTB/EPTB)	χ^2^ = 6.9	0.009	**
HIV status	χ^2^ = 1.51	0.220	ns
Previous TB	χ^2^ = 0.65	0.720	ns
Resistance type	χ^2^ = 0.59	0.810	ns
DR-TB subtype	χ^2^ = 4.15	0.044	*
Social history	χ^2^ = 1.16	0.280	ns

Notes: ns = not significant; * *p* < 0.05; ** *p* < 0.01; *** *p* < 0.001.

**Table 3:** Multivariable logistic regression analysis of predictors of treatment success.

Predictor	Adj. OR	95% CI	*p*-Value	Sig.
Age (per year)	0.98	0.963–0.998	0.026	*
PTB vs. EPTB	2.86	1.23–6.65	0.015	*
Female (vs. Male)	0.73	0.41–1.30	0.289	ns
HIV positive (vs. Neg.)	1.27	0.76–2.13	0.367	ns
Retreated (vs. New)	0.92	0.54–1.55	0.745	ns
Employed (Govt/Private)	0.93	0.41–2.09	0.863	ns
Social history (any)	0.72	0.41–1.26	0.245	ns
Poly-resistance (vs. MONO)	0.92	0.45–1.89	0.819	ns
DR-TB group (MDR+/INH)	–	–	>0.05	ns

Notes: ns = not significant; * = significant; OR = odds ratio; CI = confidence interval.

## Data Availability

Data can be requested from the corresponding author.

## Abbreviations

The following abbreviations are used in this manuscript:

ART	Antiretroviral Therapy
aOR	Adjusted Odds Ratio
CI	Confidence Interval
DoH	Department of Health (South Africa)
DR-TB	Drug-Resistant Tuberculosis
EPTB	Extrapulmonary Tuberculosis
GIS	Geographic Information Systems
HIV	Human Immunodeficiency Virus
IQR	Interquartile Range
LTFU	Lost to Follow-Up
MDR-TB	Multidrug-Resistant Tuberculosis
OR	Odds Ratio
PTB	Pulmonary Tuberculosis
RR	Rifampicin-Resistant
SD	Standard Deviation
TB	Tuberculosis
VIF	Variance Inflation Factor
WHO	World Health Organization
XDR-TB	Extensively Drug-Resistant Tuberculosis
